# Pilot study on neonicotinoids in Finnish waterbirds: no detectable concentrations in common goldeneye (*Bucephala clangula*) plasma

**DOI:** 10.1007/s11356-024-35197-3

**Published:** 2024-10-03

**Authors:** Amalie V. Ask, Pilar Gómez-Ramírez, Veerle L. B. Jaspers, José Fenoll, Juana Cava, Farshad S. Vakili, Prescillia Lemesle, Tapio Eeva, Aurélie Davranche, Sanna Koivisto, Martin Hansen, Céline Arzel

**Affiliations:** 1 Department of Biology, University of Turku, Vesilinnantie 5, 20014 Turku, Finland; 2 Area of Toxicology, Faculty of Veterinary Medicine, University of Murcia, Campus Espinardo, 30100 Murcia, Spain; 3 Department of Biology, Norwegian University of Science and Technology, 7491 Trondheim, Norway; 4 Instituto Murciano de Investigación y Desarrollo Agrario y Alimentario, IMIDA, 30150 Murcia, Spain; 5 Lammi Biological Station, University of Helsinki, 16900 Lammi, Finland; 6 Department of Biology, University of Angers, 49045 Angers, France; 7 Finnish Safety and Chemicals Agency, P.O. Box 66, 00521 Helsinki, Finland; 8 Department of Environmental Science, Aarhus University, 4000 Roskilde, Denmark

**Keywords:** Plant protection product, Veterinary medicine, Biocide, Duck, Biomonitoring, Plasma

## Abstract

Neonicotinoids have been detected in farmland-associated birds and exposure to these insecticides has been linked to adverse effects. Even though neonicotinoids are mobile and persistent and have been detected in surface waters and aquatic invertebrates, there is a considerable lack of knowledge on their occurrence in waterbirds. Here we investigated the occurrence of seven neonicotinoids and some of their transformation products (imidacloprid, thiacloprid, thiamethoxam, acetamiprid, clothianidin, dinotefuran, nitenpyram, 6-chloronicotinic acid, hydroxy-imidacloprid, imidacloprid-urea, imidacloprid-olefin, thiamethoxam-urea, thiacloprid-amide, acetamiprid-acetate, and acetamiprid-desmethyl) in blood plasma of 51 incubating female common goldeneyes (*Bucephala clangula*). We collected samples from five different regions from southern to northern Finland encompassing rural and urban settings in coastal and inland areas. Surprisingly, none of the targeted neonicotinoids was found above the limit of detection in any of the samples. As neonicotinoid concentrations in wild birds can be very low, a likely reason for the nil results is that the LODs were too high; this and other possible reasons for the lack of detection of neonicotinoids in the goldeneyes are discussed. Our results suggest that neonicotinoid exposure in their breeding areas is currently not of major concern to female goldeneyes in Finland. Even though this study did not find any immediate danger of neonicotinoids to goldeneyes, further studies including surface water, aquatic invertebrates, and other bird species could elucidate potential indirect food chain effects.

**Supplementary Information:**

The online version contains supplementary material available at 10.1007/s11356-024-35197-3.

## Introduction

Neonicotinoids are broad-spectrum insecticides which are extensively used in agriculture, veterinary medicine, and as biocides to control pests (Jeschke et al. 2011). While much of the scientific concern regarding these contaminants has been about their deleterious effects on honeybees (*Apis mellifera*) and other pollinators (see review by Dirilgen et al. 2023), neonicotinoids have also been detected in several bird species (e.g., Anderson et al. 2023; Byholm et al. 2018; Distefano et al. 2022). As neonicotinoids are persistent chemicals (Jones et al. 2014) with moderate to high water solubility (Bonmatin et al. 2015), they are mobile in the environment and have, for example, been detected in wetlands and surface waters worldwide (Casillas et al. 2022; Mehtonen et al. 2023; Morrissey et al. 2015; Perkins et al. 2021). This mobility in the environment means that bird species not directly associated with farmland are also at risk for exposure to neonicotinoids. Indeed, imidacloprid, thiacloprid, thiamethoxam, acetamiprid, and clothianidin, for instance, were detected in feathers of fledglings of two seabird species in the Mediterranean (Distefano et al. 2022) and imidacloprid was detected in 36% of plasma samples collected from different terrestrial bird species sampled in non-agricultural regions in North America (Anderson et al. 2023).

Bird populations worldwide are declining and one of the numerous drivers behind these declines is pollution (BirdLife International 2022), including neonicotinoid insecticides in agricultural environments (Hallmann et al. 2014; Li et al. 2020). Following neonicotinoid exposure, sub-lethal effects include altered migratory behavior and reduced ability to gain weight (Eng et al. 2019, 2017); endocrine disruption (Mohanty et al. 2017; Pandey and Mohanty 2015); delayed egg laying start and reduced clutch sizes (Lopez-Antia et al. 2015); lower fertilization rate, smaller egg sizes, and reduced chick survival (Lopez-Antia et al. 2013); reduced sperm density (Humann-Guilleminot et al. 2019b); and developmental toxicity (Gao et al. 2016; Wang et al. 2016).

Following the trends seen globally, many diving and dabbling duck populations have declined in Finland (Fox et al. 2015). While the usage of imidacloprid (Reg. (EU) 2018/783-4-5), thiacloprid (Reg. (EU) 2020/23), thiamethoxam (Reg. (EU) 2018/785), and clothianidin (Reg. (EU) 2018/784) in plant protection products (PPPs) in the European Union (EU) has been banned, emergency authorizations to keep using these neonicotinoids on outdoor agricultural land have been granted in Finland (European Food Safety Authority 2021). PPPs containing acetamiprid are currently permitted to use in products for agriculture, forestry, and households in Finland (KemiDigi 2024a, 2024b, 2022a, 2022b). Additionally, imidacloprid and dinotefuran are used in veterinary medicine products as ectoparasiticides in Finland (e.g., Bayvantic® Vet, Advocate®, and Vectra 3D®), and recent research suggests that such ectoparasiticides are a likely source for a considerable portion of imidacloprid detected in the aquatic environment based on studies in the UK (Perkins et al. 2024, 2021; Perkins and Goulson 2023). Finally, imidacloprid, thiamethoxam, clothianidin, and dinotefuran are used as biocides in Finland (KemiDigi 2024c). Altogether, despite neonicotinoids being under regulatory obligations, they remain widely used in Finland. A study on imidacloprid, thiacloprid, and acetamiprid in honey buzzards (*Pernis apivorus*) from western Finland detected neonicotinoids in 80% (8/10) of the samples (Byholm et al. 2018), and in 2021, imidacloprid, thiacloprid, thiamethoxam, acetamiprid, and traces of clothianidin were detected in some Finnish agricultural surface waters (SYKE 2024). Yet, no investigation has been published regarding the possible exposure of Finnish waterbirds to these contaminants.

To assess whether Finnish waterbirds might be exposed to neonicotinoids, we selected the common goldeneye (*Bucephala clangula*, hereafter goldeneye) as our model species as it is a common diving duck found across Finland, it readily nests in nest boxes close to lakes, rivers, and the sea (Pöysä et al. 1999) and has been monitored over decades in Finland by bird-ringers (see, e.g., Pöysä et al. 2017). Only the female goldeneye incubates the eggs and she forages daily throughout the circa 30-day long incubation period (Zicus and Hennes 1993), feeding primarily on aquatic invertebrates (Eadie and Keast 1982). Neonicotinoids have been detected in aquatic invertebrates (Crayton et al. 2020; Roodt et al. 2023); thus, goldeneyes might be exposed to neonicotinoids either directly through the water or through their diet. The aim of this pilot study is to assess the exposure to neonicotinoids in incubating female goldeneyes from different regions of Finland.

## Materials and methods

### Fieldwork locations

**Fig. 1:**
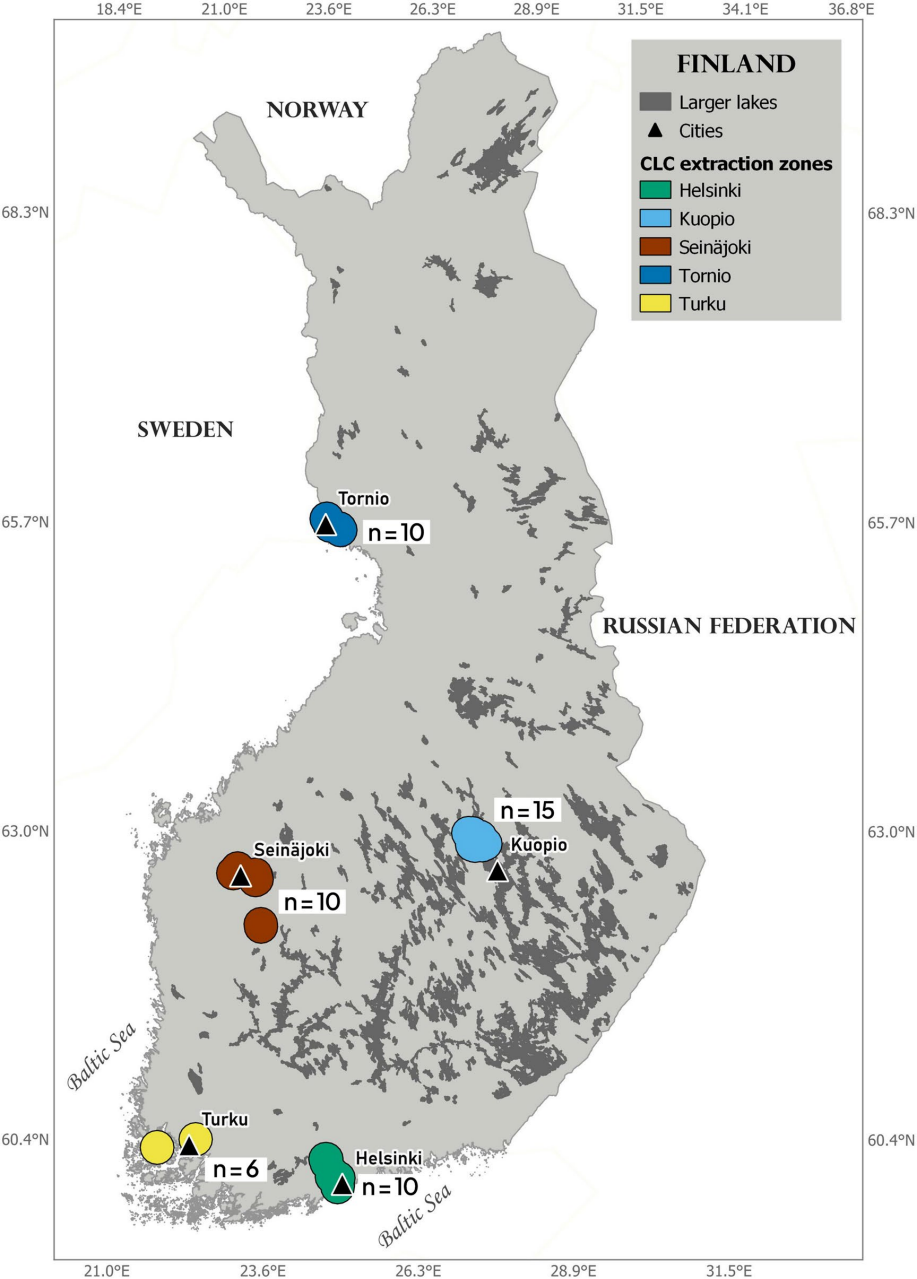
Locations of the five study regions where common goldeneye nests of incubating females were sampled in 2022. The nearest cities are identified with black triangles. “CLC extraction zones” delimit the buffer areas from which we extracted the CORINE Land Cover (2018) information for each region

Incubating goldeneye females (*n* = 51) were caught in their nest boxes from five different locations in May–June 2022 from southern to northern Finland: Turku (*n* = 6), Helsinki (*n* = 10), Seinäjoki (*n* = 10), Kuopio (*n* = 15), and Tornio (*n* = 10) (Fig. 1). Table 1 shows the primary land uses for each of the five regions based on the CORINE Land Cover (CLC) dataset from 2018 (European Environment Agency 2018). We were unable to find data on how far female goldeneyes travel to forage during incubation and, therefore, we used a buffer of 16 km for extracting the CLC as that is the maximum observed distance a Finnish female goldeneye travelled with her brood (Pentti Runko, pers. comm.). In the Supplementary Material (Table S1), we show the proportion of utilized agricultural area for the municipalities where we sampled the goldeneyes and the main crops grown there with indications when neonicotinoids may have been applied. However as spatial data on neonicotinoid use is not publicly available, we emphasize that we do not know if neonicotinoids were actually used on the crops.

**Table 1:** Proportions of each studied area covered by each category of the CORINE Land Cover (CLC) mapping approach

CLC category	Helsinki (%)	Kuopio (%)	Tornio (%)	Turku (%)	Seinäjoki (%)
Broad-leaved forest	1.15	1.27	0.88	0.07	0.00
Coniferous forest	8.44	17.31	6.79	18.14	48.14
Discontinuous urban fabric	10.91	0.77	2.70	5.57	1.72
Dump sites	0.20	1.01	0.24	0.08	0.02
Green urban areas	1.17	0.02	0.00	0.41	0.08
Industrial or commercial units	3.08	0.08	0.70	1.35	0.48
Land principally occupied by agriculture, with significant areas of natural vegetation	3.54	6.42	2.21	8.06	5.10
Mixed forest	15.38	42.18	21.49	3.53	12.09
Non-irrigated arable land	7.07	8.48	2.33	8.90	15.83
Peatbogs	0.03	0.49	2.38	0.06	5.58
Sea and ocean	44.63	0.00	53.20	52.08	0.00
Transitional woodland-shrub	0.85	6.56	3.34	0.69	7.93
Water bodies	1.64	14.74	0.35	0.18	2.77
***Other natural areas***					
Salt marshes	0.19	0.00	0.08	0.16	0.00
Water courses	0.00	0.00	1.98	0.02	0.02
Natural grasslands	0.00	0.00	0.01	0.00	0.00
Moors and heathland	0.00	0.00	< 0.01	0.00	0.00
Estuaries	0.00	0.00	0.42	0.00	0.00
Coastal lagoons	0.00	0.00	0.03	0.00	0.00
Inland marshes	0.00	0.36	0.02	0.00	0.12
***Other infrastructure and anthropogenic***					
Road and rail networks and associated land	0.19	0.00	0.06	0.00	0.00
Port areas	0.00	0.00	0.06	0.22	0.00
Airports	0.53	0.00	0.06	0.09	0.03
Construction sites	0.14	0.00	0.00	0.04	0.00
Mineral extraction sites	0.11	0.22	0.26	0.08	0.01
Sport and leisure facilities	0.72	0.09	0.10	0.27	0.05
***Other agriculture***					
Pastures	0.02	0.00	0.29	0.00	0.04
Fruit trees and berry plantations	0.01	0.00	0.00	0.00	0.00

### Blood sampling

Blood was collected from the brachial vein and kept cold (approximately 4 °C) and dark until centrifugation within 8 h (2500 rpm, 10 min). An aliquot of the plasma was transferred to an Eppendorf tube and frozen at − 20 °C while in the field stations and transferred to a − 80 °C freezer when returning to the University of Turku. A total of nine field blanks of water (molecular biology grade, Thermo Fisher Scientific, USA) were taken across the five locations. The plasma and blank samples were sent on dry ice to the University of Valencia, Spain, for sample preparation.

### Chemicals and reagents

LC/MS grade acetonitrile (Optima) was purchased from Thermo Fisher Scientific (USA) and ultrapure water was obtained using the Ecomatic system (type II analytical grade water) from Wasserlab (Spain). Reagent grade NaCl was purchased from Honeywell (USA). The authentic standards of imidacloprid, thiamethoxam, thiacloprid, acetamiprid, clothianidin, dinotefuran, and nitenpyram were all purchased from Sigma-Aldrich (Steinheim, Germany).

### Sample preparation

Plasma samples were prepared following a modification of the method described by Martínez et al. (2022). Briefly, 100 µL of plasma was pipetted into a 1.5 mL Eppendorf tube and 1 mL acetonitrile:water (1:1 v/v) was added. The tubes were then vortexed for 10 s before being placed in an ultrasound bath (VWR, USA) for 20 min (ice was added to keep the water temperature low). Then 100 mg of NaCl was added to each tube, which was then vortexed for 5 min. The tubes were then centrifuged for 5 min at 10,000 rpm at 5 °C (3K15, Sigma). Finally, the supernatant was collected with a syringe (NORM-JECT, Henke Sass Wolf) and filtered through a 0.2-µm nylon filter (Econo Filter, Captiva, Agilent Technologies, USA) into an autosampler vial with glass insert. There was enough supernatant for two vials per sample.

### HPLC–MS analysis

The samples were sent to the Instituto Murciano de Investigación y Desarrollo Agrario y Medioambiental (IMIDA) in Murcia, Spain, for instrumental analysis of seven neonicotinoids (imidacloprid, thiamethoxam, thiacloprid, acetamiprid, clothianidin, dinotefuran, and nitenpyram) and some of their transformation products (6-chloronicotinic acid, hydroxy-imidacloprid, imidacloprid-urea, imidacloprid-olefin, thiamethoxam-urea, thiacloprid-amide, acetamiprid-acetate, and acetamiprid-desmethyl). We followed the method described in Martínez et al. (2022), and it will thus only be briefly summarized here. Samples were analyzed with high-pressure liquid chromatography (HPLC; Agilent Series 1200, Agilent Technologies) containing a reverse phase C8 analytical column (150 × 4.6 mm, 5 µm, ZORBAX Eclipse XDB-C8) hyphenated to a triple quadrupole mass spectrometer (MS; G6410A, Agilent Technologies). Ionization was achieved through electrospray in positive mode. The mobile phases were acetonitrile (A) and 0.1% formic acid in water (B). The gradient program began with 10% A (for 5 min), linear gradient to 100% A (35 min), followed by 10 min post-run time with 10% A. Flow rate was kept at 0.7 mL min^−1^ and sample injection volume was 10 µL. Fragmentor voltages and collision energies applied to the compounds, along with their retention times and their optimized SRM transitions, are described by Klaas-Fábregas et al. (2024).

### Quality assurance/quality control (QA/QC)

Calibration curves of the seven parent neonicotinoids were made with concentrations 0.625–160 ng mL^−1^. To validate the method, plasma from healthy hens (*Gallus gallus domesticus*) was obtained from the Laboratory Animals section from the University of Murcia (authorization code CEEA 177/2015). The plasma samples (100 µL per sample) were spiked with the seven parent neonicotinoids in triplicate (in concentrations from 0.5 to 20 ng mL^−1^). Recoveries ranged from 68 to 137%, except for nitenpyram (52% in all spiked samples) and all showed good coefficients of determination (R^2^ > 0.99). The limits of quantification (LOQs) were 5 ng mL^−1^ for dinotefuran, 2 ng mL^−1^ for nitenpyram, and 0.5 ng mL^−1^ for thiamethoxam, clothianidin, imidacloprid, acetamiprid, and thiacloprid. The limits of detection (LODs) were 1.67 ng mL^−1^ for dinotefuran, 0.67 ng mL^−1^ for nitenpyram, and 0.17 ng mL^−1^ for thiamethoxam, clothianidin, imidacloprid, acetamiprid, and thiacloprid. To account for instrumental drift and carry-over, a calibration standard and two solvent blank samples were injected regularly (after every 10th to 16th plasma sample). Nine field blanks and ten laboratory blanks were included to account for contamination from field and lab equipment—none of these blanks had detectable concentrations of the target analytes.

## Results and discussion

None of the targeted neonicotinoids was found above the limits of detection in any of the goldeneye samples. Given that neonicotinoids have previously been detected in several different genera of birds—including raptors from Finland—we expected to detect some neonicotinoids. Below, we discuss possible reasons for why none of the targeted neonicotinoids was detected.

A contributing factor to our nil results might be the sensitivity of the method, as neonicotinoid concentrations detected in plasma of wild birds can be low. Hao et al. (2018), for instance, found a maximum concentration of thiamethoxam of 0.0337 ng mL^−1^ in white-crowned sparrow (*Zonotrichia leucophrys*) plasma. In our study, the LOD for this compound is 0.17 ng mL^−1^. Thus, we cannot exclude the possibility that thiamethoxam residues were in the plasma, but at too low concentrations to be detected. The same applies to the other neonicotinoids, and especially to dinotefuran and nitenpyram which have LODs of 1.67 and 0.67 ng mL^−1^, respectively, in our study, which might have precluded detection of these neonicotinoids.

Even though neonicotinoids in PPPs have been restricted in Finland, they are still used in a range of settings as described earlier; thus, current use of neonicotinoids in Finland cannot explain the non-detection in the goldeneye samples. Indeed, despite the restrictions, PPPs containing imidacloprid and thiamethoxam were given emergency authorization for use in 2022 and thiacloprid and clothianidin were given emergency authorization for use in 2021 (European Commission 2024). Furthermore, in 2023, imidacloprid was detected in stormwater from a residential area in the Turku region at 0.034 µg L^−1^ (NonHazCity3, 2024), which the authors posit is from biocides and/or veterinary medicine products—highlighting that non-agricultural sources must also be considered. However, an exception is nitenpyram which, in Finland, is not used as a pesticide or biocide and its sale permission in veterinary medicine products has been cancelled.

The continued use of neonicotinoids in Finland, however, does not mean that all areas of Finland are contaminated or that contamination is spatially uniform. We collected our samples from a diverse range of areas (inland and coastal, agricultural and more urbanized), in order to capture a larger set of possible use cases of neonicotinoids (see Fig. 1 and Table 1). In addition, our samples from the Seinäjoki region were collected close to the same area where Byholm et al. (2018) detected neonicotinoids (thiacloprid and imidacloprid) in whole blood from honey buzzards in 2013. One likely reason why Byholm and colleagues detected neonicotinoids and we did not is that the LOD for thiacloprid and imidacloprid in our study is higher (0.17 ng mL^−1^) than the maximum concentrations of 31 and 8.9 pg mL^−1^ for thiacloprid and imidacloprid, respectively, found in the honey buzzards (Byholm et al. 2018). A further reason for the differing results is likely the combination of the different diets and exposure pathways of honey buzzards and goldeneyes together with the different regulatory landscape of neonicotinoids in PPPs during our respective sampling campaigns (2013 and 2022). Byholm and colleagues found that presence of neonicotinoids in nestlings matched with presence of turnip and rape fields within 2 and 5 km of the nests, and they sampled the honey buzzards right before the first set of EU restrictions on imidacloprid, thiamethoxam, and clothianidin came into effect (Reg. (EU) No 485/2013). Thus, it is reasonable to assume that PPPs containing neonicotinoids were the main exposure source for the honey buzzards. Honey buzzards feed on larvae of wasps and bumblebees (Byholm et al. 2018) and are thus in more direct contact with potentially treated agricultural lands. Goldeneyes, on the other hand, feed on aquatic invertebrates and—regardless of the source of neonicotinoids—the major exposure vectors are water and diet. In both cases, dilution of the neonicotinoids is most likely the primary reason why we did not detect any neonicotinoids in our samples (see, e.g., Schaafsma et al. 2019). Indeed, the Finnish Environment Institute monitors five neonicotinoids in agricultural surface waters, and in 2022, only imidacloprid and acetamiprid were measured above the LOQ (2/79 and 1/78 samples, respectively) at concentrations of 0.15 and 0.04 µg L^−1^ for imidacloprid and 0.01 µg L^−1^ for acetamiprid (open data accessible through registration at SYKE 2024). These concentrations fall towards the lower end of the typical range of neonicotinoid concentrations in agricultural surface waters (Stehle et al. 2023).

A further consideration is the choice of sample matrix. In our study, we chose plasma to assess goldeneye exposure to neonicotinoids. Neonicotinoids have been detected in plasma from multiple different bird species (e.g., Anderson et al. 2023; Hao et al. 2018; Humann-Guilleminot et al. 2021) and it was thus expected that recent exposure to neonicotinoids in the breeding areas would be captured in the plasma, as goldeneyes do forage daily throughout the incubation period (Zicus and Hennes 1993). However, plasma is not well-suited for identifying past exposure as imidacloprid, thiamethoxam, and clothianidin are rapidly metabolized (within hours) (Bean et al. 2019; Pan et al. 2022; Roy et al. 2020), with the assumption that the other neonicotinoids display approximately the same toxicokinetics. Thus, by including feathers (Humann-Guilleminot et al. 2019a), for example, as well as plasma samples, we would have captured a longer exposure window and we recommend that future studies investigate the suitability of feathers for monitoring neonicotinoids.

The time of sampling within the year should also be considered: a study on terrestrial birds from non-agricultural regions in the USA found higher rates of occurrence of neonicotinoids in birds in spring and fall compared to summer and winter (Anderson et al. 2023), which the authors posit is due to the sowing of neonicotinoid-coated seeds in nearby croplands and the neonicotinoids’ subsequent mobility in the environment. The two PPPs which were granted emergency authorization in 2022 could be legally applied from mid-February to mid-June (European Commission 2024). Our sampling campaign was May 15th–June 17th, thus covering the legal application period of these two PPPs. Unfortunately, we could not access information on application periods for PPPs containing acetamiprid. Regarding possible seasonal variation of neonicotinoids derived from veterinary medicine products and biocides, a study on wastewater in the USA found a seasonal variation in concentrations of clothianidin in wastewater effluents, but no such variation in imidacloprid and thiamethoxam concentrations (Webb et al. 2021). This is in line with the results of Perkins and Goulson (2023) who found that 34% of surveyed pet owners use flea treatment year-round. Thus, we do not consider seasonal variation to be a determining factor for why imidacloprid and thiamethoxam were not detected in any of the goldeneye samples and instead dilution in the aquatic environment is a likely reason for the non-detection in the goldeneyes.

Lastly, our study aimed to assess whether goldeneyes are exposed to neonicotinoids and, thus, potentially directly affected by them. However, there is evidence that insectivorous birds are affected by pesticides, not necessarily through direct toxic action, but through pesticides’ depletion of insect populations and, consequently, the insectivorous birds’ prey populations (Møller et al. 2021). Our study design did not incorporate the study of populations of aquatic invertebrates which goldeneyes forage on, and it is therefore not suited for answering whether goldeneyes are indirectly affected by neonicotinoids in the manner of reducing population abundances of their prey species.

In conclusion, our results suggest that neonicotinoids do not appear to be of direct concern to breeding female goldeneyes in Finland. However, there might be indirect food chain effects of neonicotinoids and we strongly encourage future research efforts to investigate the occurrence of neonicotinoids in a larger set of agricultural and urban surface waters, in aquatic invertebrates, and in other bird species.

## Supplementary information

Below is the link to the electronic supplementary material.

Supplementary file1 (DOCX 29 kb)

## Data Availability

All data is presented in the manuscript.
