# Supplementary material for: Pilot study on neonicotinoids in Finnish waterbirds: no detectable concentrations in common goldeneye (Bucephala clangula) plasma
Source: Environ Sci Pollut Res Int. 2024 Oct 3;31(52):61950–8. doi: 10.1007/s11356-024-35197-3 (PMC11541392; doi:10.1007/s11356-024-35197-3)
Supplement: Supplementary file 1 — Supplementary file1 (DOCX 29 kb) [file 11356_2024_35197_MOESM1_ESM.docx]

**Supplementary Material**

**for**

**Pilot study on neonicotinoids in Finnish waterbirds: no detectable concentrations in common goldeneye (*Bucephala clangula*) plasma**

Amalie V. Ask^1^*, Pilar Gómez-Ramírez^2^, Veerle L. B. Jaspers^3^, José Fenoll^4^, Juana Cava^4^, Farshad S. Vakili^1^, Prescillia Lemesle^3^, Tapio Eeva^1^, Aurélie Davranche^5,6^, Sanna Koivisto^7^, Martin Hansen^8^, and Céline Arzel^1^

^1^Department of Biology, FI-20014, University of Turku, Finland

^2^Area of Toxicology, Faculty of Veterinary Medicine, ES-30100, University of Murcia, Campus Espinardo, Murcia, Spain

^3^Department of Biology, Norwegian University of Science and Technology, NO-7491 Trondheim, Norway

^4^Instituto Murciano de Investigación y Desarrollo Agrario y Alimentario, IMIDA, ES-30150 Murcia, Spain

^5^Lammi Biological Station, FI-16900, University of Helsinki, Finland

^6^Department of Biology, FR-49045, University of Angers, France

^7^Finnish Safety and Chemicals Agency, P.O. Box 66, FI-00521 Helsinki

^8^Department of Environmental Science, Aarhus University, DK-4000 Roskilde, Denmark

*Corresponding author, [amalie.ask@utu.fi](mailto:amalie.ask@utu.fi)

**Table S1**: The main crops produced in 2022 in the municipalities where the goldeneyes were sampled (sample sizes in parentheses). The data was retrieved from the Natural Resources Institute Finland (Luke, 2023) and modified to show the percentage of crop type out of the total utilized agricultural area (UAA, in hectare) per municipality. Crop types in bold are those where neonicotinoids *might* be used based on application/authorization permits, but pesticide use data is not accessible on the scale of municipalities. Note that only registered farms are included in the statistics, and this table may thus be underestimating the actual proportion of the crops. “-“ refers to cases where either the information is missing from the database or too few farms produced the crop and the data is thus confidential.

|  | Turku | | Helsinki | | | | Kuopio | Seinäjoki | | | Tornio |
| --- | --- | --- | --- | --- | --- | --- | --- | --- | --- | --- | --- |
| Municipality | Turku (1) | Naantali (5) | Helsinki (4) | Espoo (1) | Vantaa (1) | Nurmijärvi (4) | Kuopio (15) | Seinäjoki (5) | Virrat (4) | Ilmajoki (1) | Tornio (10) |
| **%Fodder grassland** | 20.8 | 24.3 | 33.9 | 19.6 | 19.3 | 21.1 | 55.7 | 21.6 | 51.5 | 20.2 | 80.8 |
| %Spring wheat | 18.7 | 15.2 | 14.3 | 28.4 | 25.3 | 19.9 | 1.5 | 8.0 | 2.1 | 6.4 | - |
| %Oats | 16.1 | 11.5 | - | 11.6 | 10.6 | 11.5 | 4.9 | 21.8 | 14.3 | 13.9 | 0.6 |
| %Feed barley | 2.5 | 6.4 | 15.1 | - | 8.2 | 7.0 | 22.5 | 26.5 | 13.5 | 37.4 | 2.9 |
| %Rye | 0.5 | 2.0 | 0.0 | - | 2.0 | 1.7 | 0.1 | 1.1 | - | 1.9 | - |
| **%Peas** | 3.5 | 0.9 | - | 2.7 | - | 2.5 | 0.4 | 2.3 | 0.7 | 2.4 | 0.2 |
| **%Turnip rape** | - | 1.3 | 6.5 | - | 0.9 | 1.8 | 1.5 | 2.3 | 2.0 | 2.5 | - |
| **%Rape** | 2.2 | 1.2 | - | - | 1.6 | 1.2 | - | - | - | 0.2 | - |
| **%Potatoes** | 0.5 | 3.7 | - | 0.0 | 0.0 | 0.0 | 0.1 | 0.9 | 0.0 | 0.4 | 0.0 |
| **%Sugar beet** | - | - | - | - | - | - | - | - | - | - | - |
| **%Horticultural crops** | 1.3 | 2.2 | - | 0.6 | 0.4 | 0.8 | 0.9 | 0.1 | 0.1 | - | - |
| **%Permanent crops** | 0.2 | 0.3 | - | - | - | 0.1 | 0.4 | 0.0 | 0.2 | 0.4 | - |
| %Whole crop cereals | - | - | - | - | - | - | 1.3 | 0.8 | 1.2 | 1.4 | 5.3 |
| %Other crops | 2.2 | 0.7 | - | - | 1.2 | 0.9 | 0.3 | 0.4 | 0.0 | 0.5 | 0.1 |
| %Grassland | 1.3 | 1.6 | 2.3 | 0.5 | 0.2 | 0.3 | 0.5 | 0.2 | 0.7 | 0.2 | 1.3 |
| Municipality area (ha)* | 24 563 | 31 258 | 21 442 | 31 235 | 23 838 | 36 190 | 324 174 | 143 177 | 116 263 | 57 674 | 118 871 |
| Total UAA (ha) | 3987 | 4519 | 992 | 1728 | 3496 | 10 983 | 33 068 | 32 355 | 8705 | 18 081 | 7641 |
| %UAA | 16.2 | 14.5 | 4.6 | 5.5 | 14.7 | 30.3 | 10.2 | 22.6 | 7.5 | 31.4 | 6.4 |

*Only the land area

**References**

Luke, 2023. Utilised Agricultural Area 2022 [WWW Document]. Natural Resources Institute Finland. URL https://www.luke.fi/en/statistics/utilised-agricultural-area/utilised-agricultural-area-2022 (accessed 8.30.24).
